# Improved cognitive impairments by silencing DMP1 via enhancing the proliferation of neural progenitor cell in Alzheimer‐like mice

**DOI:** 10.1111/acel.13601

**Published:** 2022-04-02

**Authors:** Huimin Zhao, Jie Wei, Yanan Du, Peipei Chen, Xiaoquan Liu, Haochen Liu, for the Alzheimer’s Disease Neuroimaging Initiative

**Affiliations:** ^1^ 56651 Center of Drug Metabolism and Pharmacokinetics China Pharmaceutical University Nanjing China

**Keywords:** Alzheimer's disease, DMP1, neural progenitor cell, proliferation

## Abstract

Alzheimer's disease (AD) is age‐related progressive neurological dysfunction. Limited clinical benefits for current treatments indicate an urgent need for novel therapeutic strategies. Previous transcriptomic analysis showed that DMP1 expression level was increased in AD model animals whereas it can induce cell‐cycle arrest in several cell lines. However, whether the cell‐cycle arrest of neural progenitor cell induced by DMP1 affects cognitive function in Alzheimer‐like mice still remains unknown. The objective of our study is to explore the issue. We found that DMP1 is correlated with cognitive function based on the clinical genomic analysis of ADNI database. The negative role of DMP1 on neural progenitor cell (NPC) proliferation was revealed by silencing and overexpressing DMP1 in vitro. Furthermore, silencing DMP1 could increase the number of NPCs and improve cognitive function in Alzheimer‐like mice, through decreasing P53 and P21 levels, which suggested that DMP1‐induced cell‐cycle arrest could influence cognitive function.

## Graphical abstract

Neural progenitor cells (NPC) emerged their roles in endogenous regenerating neurons for Alzheimer's disease (AD) treatments. Clinical genomics data and AD animal model experiments suggested that DMP1 was related to cognitive function and NPC proliferation. Silencing DMP1 could improve cognitive function through increase newborn NPC and neurons in AD animal model. Data demonstrated the potential role of DMP1 for AD treatment by stimulating NPC proliferation.

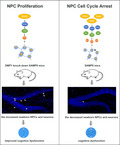

**Abbreviations**
ADAS‐cog: Alzheimer’s disease assessment scale
AD: Alzheimer’s disease
ADNI: Alzheimer’s Disease Neuroimaging Initiative
BrdU: 5‐Bromodeoxyuridinc
CDKs: cyclin dependent kinases
DCX: doublecortin
DG: dentate gyrus
DMP1: cyclin D‐binding myb‐like protein 1
GFP: green fluorescent protein
MCI: mild cognitive impairment
MWM: green fluorescent protein
NPC: neural progenitor cell
ROS: reactive oxygen species
SGZ: subgranular region of dentate gyrus
SNP: single nucleotide polymorphism
SVZ: subventricular zone
WGS: whole genome sequencing

## 1 INTRODUCTION

Alzheimer's disease (AD) is considered as one of the most common causes of dementia (Crous‐Bou et al., 2017). AD generally develops in aging adults associated with memory loss and cognitive impairment (Sengoku, 2020). Normally, the pathology of AD is characterized by extracellular amyloid peptides (Aβ) deposition, intracellular tangles of fibrillar phosphorylated Tau protein, neuronal loss and synapse loss (Lane et al., 2018; Pimenova et al., 2018; Vlassenko et al., 2012). Current treatments focused on targeting at neurofibrillary tangles, senile plaques, oxidative stress reduction, anti‐inflammatory, modulation of cellular calcium homeostasis and neurotransmission, has some limited effectiveness (Revi, 2020). Consequently, these issues have drawn the attention of many researchers to the importance of developing new therapies against this disease.

In recent decades, preventing neuronal loss has become more prevalent research area for finding effective treatments against AD (Donev et al., 2009; Giorgini, 2013). Previous studies suggested that therapeutic strategies for neuronal loss include neurons apoptosis inhibition and stem cell therapy (Telegina et al., 2019; Vasic et al., 2019). Yet protecting neurons from apoptosis simply lacks efficacy for cognitive impairment in some AD animal models (He et al., 2020; Kim et al., 2020; Yu et al., 2020), it is essential to highlight the role of stem cell therapy. Engrafted regeneration and endogenous regeneration are the most common methods utilized for stem cell therapy in the relevant research studies (Vasic et al., 2019). Several studies have showed the positive effect of in vivo transplantation by utilizing specific cell types in AD animal models. There were varieties of established transplantation protocols: Mouse and human embryonic stem cells were first induced into mature basal forebrain cholinergic neurons and then transplanted into the AD rat model (Moghadam et al., 2009); Human neural stem cells from fetal telencephalon were transplanted into an AD mouse brain (Lee et al., 2015); By treating specific protein extracts, transplantation of induced pluripotent stem cells derived from mouse skin fibroblasts in the 5XFAD transgenic AD mouse model was carried out (Cha et al., 2017); After transplantation into the hippocampus of AD mice, neuronal precursors were successfully differentiated into cholinergic neurons (Fujiwara et al., 2013). All these established strategies of in vivo trans‐differentiation were achieved with the improvement on learning and memory deficits in AD animal models. Although previous studies have been proposed that numerous stem cell types are pluripotent and multipotent, the issues such as its poor survival, possible pathological phenotype and low rate of neuronal differentiation after transplantation, restricted its application (Fujikawa et al., 2005; Lee et al., 2003; Liang et al., 2013; Nakaji‐Hirabayashi et al., 2013; Richards et al., 2002). While endogenous regeneration is a process of generating adult‐born neurons from neural progenitor cells (NPCs) in the specific area of brain (Zhao et al., 2008). Emerging evidence shows that this process is aberrant in AD mouse models and AD patients (Boekhoorn et al., 2006; Crews et al., 2010; Donovan et al., 2006; Ermini et al., 2008; Jin et al., 2004; Wen et al., 2004). Therefore, we wonder that whether it can be a therapeutic strategy by improving this abnormal process of AD. Furthermore, previous research suggested that stimulating the proliferation of NPCs could be an effective therapeutic strategy in cognitive decline improvement in several AD mouse models (Huang et al., 2017; Kong et al., 2015; Mao et al., 2015; Morello et al., 2018). These findings indicated that the number of new neuroblasts and immature neurons is markedly increased after inducing the proliferation of NPC, which have functional consequences for the hippocampal network (Choi et al., 2018). Among these studies, NPC proliferation could be promoted through extrinsic administration of chemical agents, growth factors (Herrán et al., 2013; Huang et al., 2017; Lee et al., 2012; Liu & Nusslock, 2018; Sachs & Caron, 2014) and physical exercise(Choi et al., 2018; van Praag et al., 1999), but the molecular basis of this process is mostly undefined. Based on previous research, we assume that the reduced proliferation of NPCs is related to its cell‐cycle dysregulation, which further results in cognitive dysfunction. Also, the mechanism involved in causing cell‐cycle arrest of NPCs in AD should be verified.

Cell‐cycle arrest is known to relate to several signaling pathways, such as PI3K/Akt, RAS, MEK/ERK, JNK, NF‐κB and NFAT. Among these pathways (Pietenpol & Stewart, 2002), the RAS pathway is abnormally activated in postmortem human AD brains (Dineley et al., 2001). In parallel, mitogenic signals from oncogenic RAS (Sreeramaneni et al., 2005) have been reported to activate the DMP1 promoter to increase the expression of Dmp1 protein and microarray studies showed that DMP1 expression level was increased in AD model animals (Hokama et al., 2014). DMP1 (cyclin D–binding myb‐like protein 1; Dmtf1) is a tumor suppressor, which can induce P53‐medicated cell‐cycle arrest by Arf‐independent and Arf‐dependent manners (Frazier et al., 2012; Inoue et al., 1999, 2007, 2016; Sugiyama et al., 2008). In another case, the DMP1/P53 signaling pathway blocked mouse fibroblasts proliferation by induction of the CDK inhibitor P21cip1/waf1 (Inoue & Sherr, 1998). Therefore, DMP1 seems to participate in the dysregulation of NPC cell cycle, resulting in the impairment of NPC proliferation capacity in AD, but mechanistically how it triggers and contributes to NPC growth suppression is not known.

In addition to existing studies, the role of DMP1 on the development of AD has not been investigated. Our research originated from the analysis of the association between the DMP1 SNP data and cognitive function. Next, the effects of DMP1 on NPCs proliferation were assessed by silencing and overexpressing DMP1. To further elucidate the potential roles of DMP1 on hippocampal NPC proliferation and cognitive function of SAMP8 mice, we evaluated the cognitive function and newborn NPC in animals. Finally, the main objective of this work was to analyze the role of Dmp1 in NPC proliferation under AD condition in vitro and in vivo. Our research provides novel insight into the future treatment in AD.

## 2 RESULTS

### 2.1 DMP1 SNPs were correlated with cognitive function in AD patients

818 subjects in ADNI cohort received whole genome sequencing. Because of the failure in quality control, 9 subjects were discarded. Hence, we did the following analysis of 809 subjects. Table S1 showed the demography information of this ADNI cohort (Supporting Information). There are three groups after grouping the subjects: AD group (*n* = 48), MCI group (*n* = 480) and control group (*n* = 281). The proportions of men in the MCI group (58%) and control group (48%) have no significant difference (*p* > 0.05). The proportion of men in the AD group (38%) was less than in MCI group (58%) and control group (48%). The subjects’ age distribution among these three groups are quite semblable.

**FIGURE 1:**
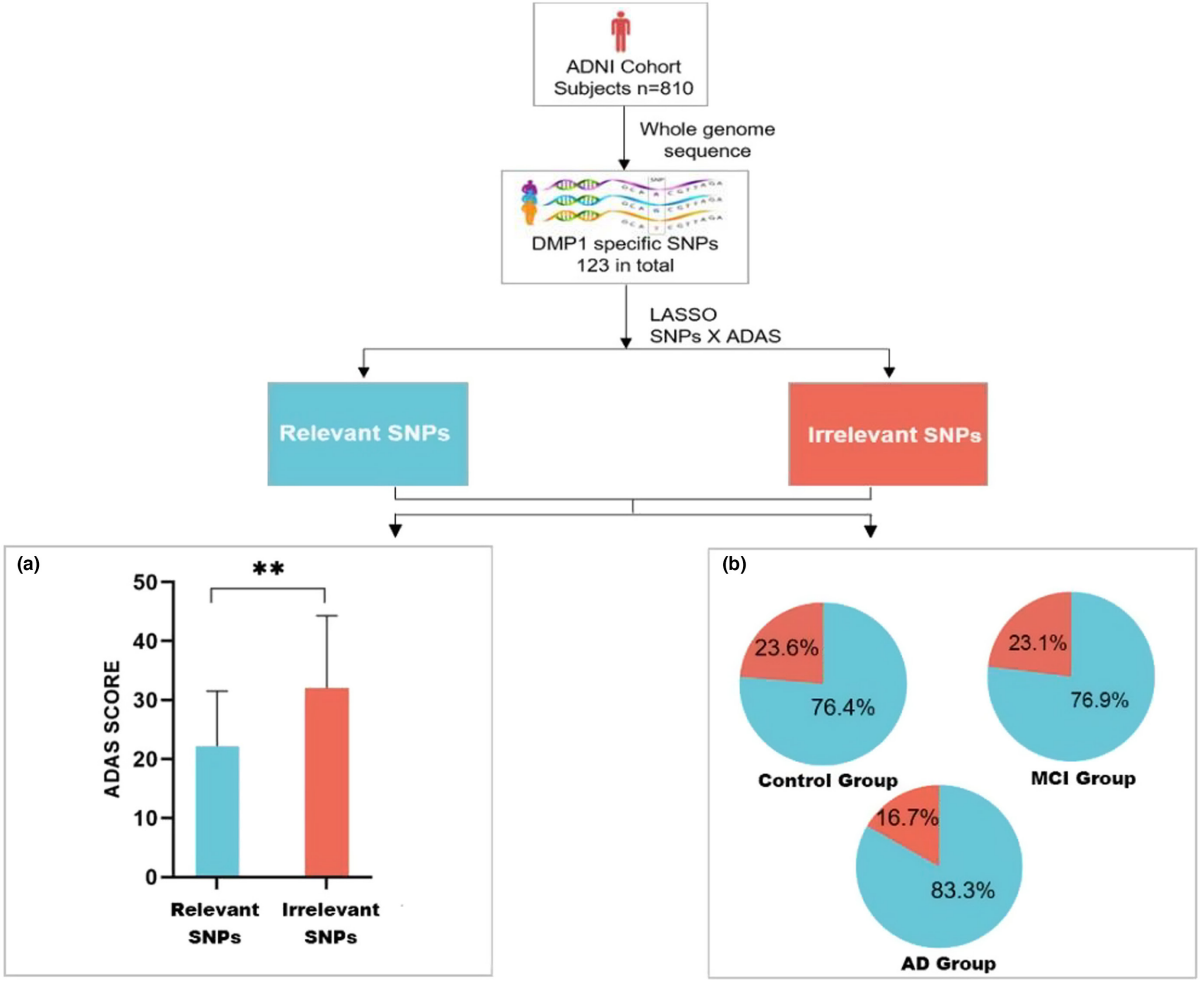
Exploration of the correlation between DMP1 specific SNPs and cognitive function. (a) The comparison of ADAS levels between the relevant SNPs cluster and the irrelevant SNPs cluster (*p* < 0.01). (b) The distribution of the relevant SNPs cluster and the irrelevant SNPs cluster among the control group, the MCI group and the AD group

We investigated the association between DMP1 SNPs and ADAS‐cog in AD patients. Firstly, the 123 DMP1 SNPs were extracted from the WGS data set. The SNPs that are too common in subjects (frequency > 90%) and rare in subjects (frequency < 5%) are excluded for avoiding potential statistical bias. Then 23 DMP1 SNPs were remained (showed in Supporting Information Table S2). Secondly, LASSO regression is used to evaluate the relevance between 23 SNPs and ADAS‐cog. According to the LASSO regression results, the SNPs were classified to four clusters: SNPs were negatively correlated with ADAS‐cog (termed as protective SNPs, *n* = 0); SNPs were positively correlated with ADAS‐cog (termed as exacerbating SNPs, *n* = 3); SNPs were both negatively and positively correlated with ADAS‐cog (termed as relevant SNPs, *n* = 31); SNPs were irrelevant to ADAS‐cog and other unstudied SNPs (termed as irrelevant SNPs, *n* = 12). To reduce statistical error, cluster that contained too few subjects (*n* < 5) was excluded. Based on this, clusters of exacerbating SNPs and protective SNPs were excluded. Then we compared the ADAS‐cog between the two clusters (shown in Figure 1a). Subjects harboring irrelevant SNPs get significantly higher ADAS‐cog level than subjects harboring exacerbating and protective SNPs (*p* < 0.01). Furthermore to investigate the impact of different DMP1 SNPs on AD risk, the MCI‐to‐AD conversion proportions in the two clusters (exacerbating and protective SNPs, irrelevant SNPs) were investigated. The pooled diagnostic odds ratio was 1.06 (95% CI: 0.63–2.49, relevant SNPs vs irrelevant SNPs, *p* > 0.05), which suggested that DMP1 SNPs may not be associated with MCI‐to‐AD conversion risk. Finally, we compared the distribution of the two clusters among the healthy individuals, the MCI patients and the AD patients, but no significant differences were found in the distribution of each cluster. These data suggested that DMP1 SNPs were correlated with cognitive function in AD patients.

### 2.2 Aβ_1–42_ exposure resulted in the cell‐cycle arrest of C17.2 neural progenitor cells and overexpression of DMP1

**FIGURE 2:**
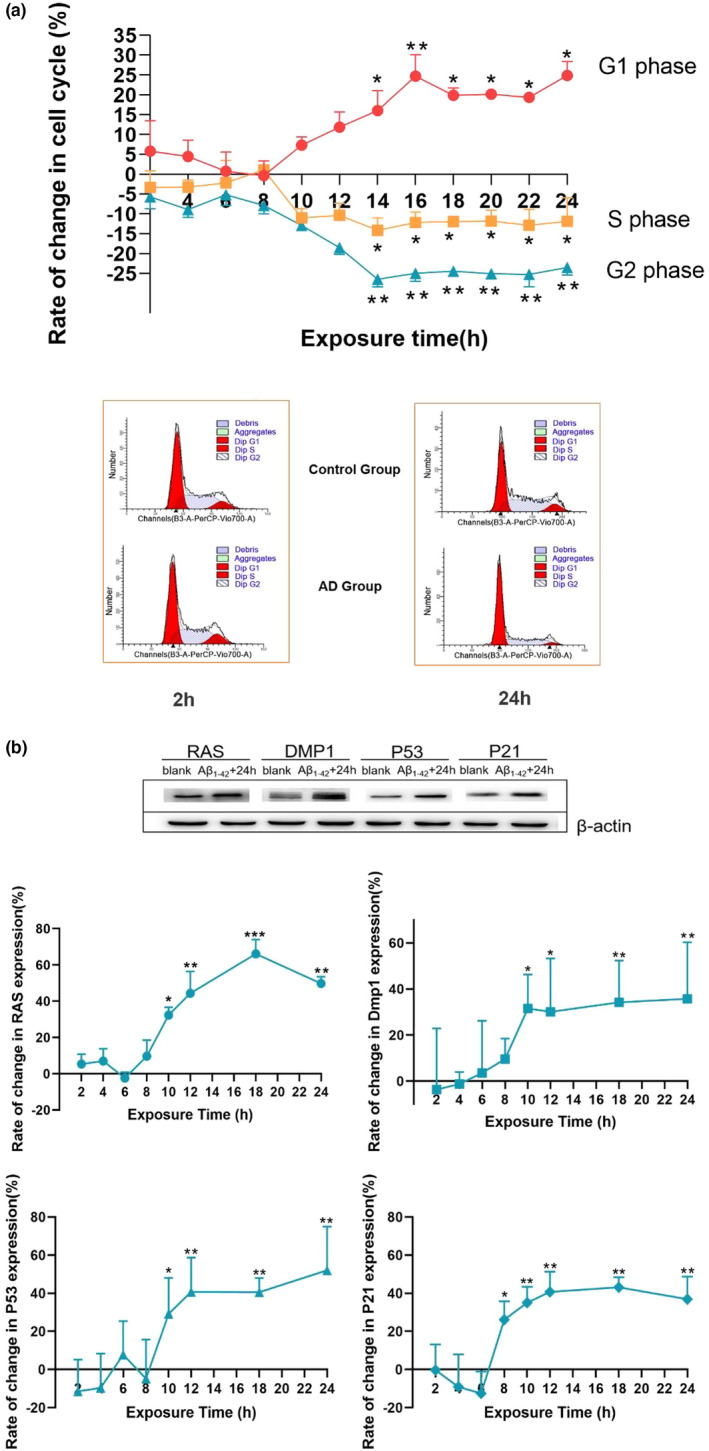
Aβ_1–42_ exposure leads to the cell‐cycle arrest of C17.2 NPCs and the increased expression of DMP1. (a) Rate of change in cell cycle of C17.2 NPCs. At the indicated time point, DNA content of the cells were determined to analyze the distribution of cell‐cycle phase. The mathematical model MODFIT was used to calculate the proportions of cells at each cell‐cycle phase. (b) Rate of change in RAS, DMP1, P53 and P21 expression at different time point. Data information: Mean ± SEM, *n* = 3 for each group, *p < 0.05, **p < 0.01, ***p < 0.001 the Aβ_1–42_ group vs the control group

To access the change of C17.2 neural progenitor cells proliferation, we divided cultures of C17.2 neural progenitor cells into two groups, one group was the normal control group; the other group was given 20 μmol/ml Aβ_1–42_ to prepare AD model in vitro. At 2, 4, 6, 8, 10, 14, 18 and 24 h, the number of cells with each cell cycle phase was quantified by flow cytometry (FCM); the expression of RAS, DMP1, P53 and P21 proteins was measured by western blot. Compared with the control group, an augmented quantity of cells is observed in G1 phase and the amount of S and G2 phase cells was decreased after 12 h Aβ_1–42_ exposure in AD group (Figure 2). These data suggested that the G1 phase arrest of C17.2 NPCs was induced by Aβ_1–42_. Furthermore, the expression of RAS, DMP1, P53 and P21 proteins was significantly higher than the control group after 10 h Aβ_1–42_ exposure (Figure 2, *p* < 0.05). These findings suggested that Aβ_1–42_ exposure resulted in the G1 phase arrest of C17.2 NPCs through increasing Dmp1 expression.

### 2.3 DMP1 knockdown improved proliferation of C17.2 neural progenitor cells

**FIGURE 3:**
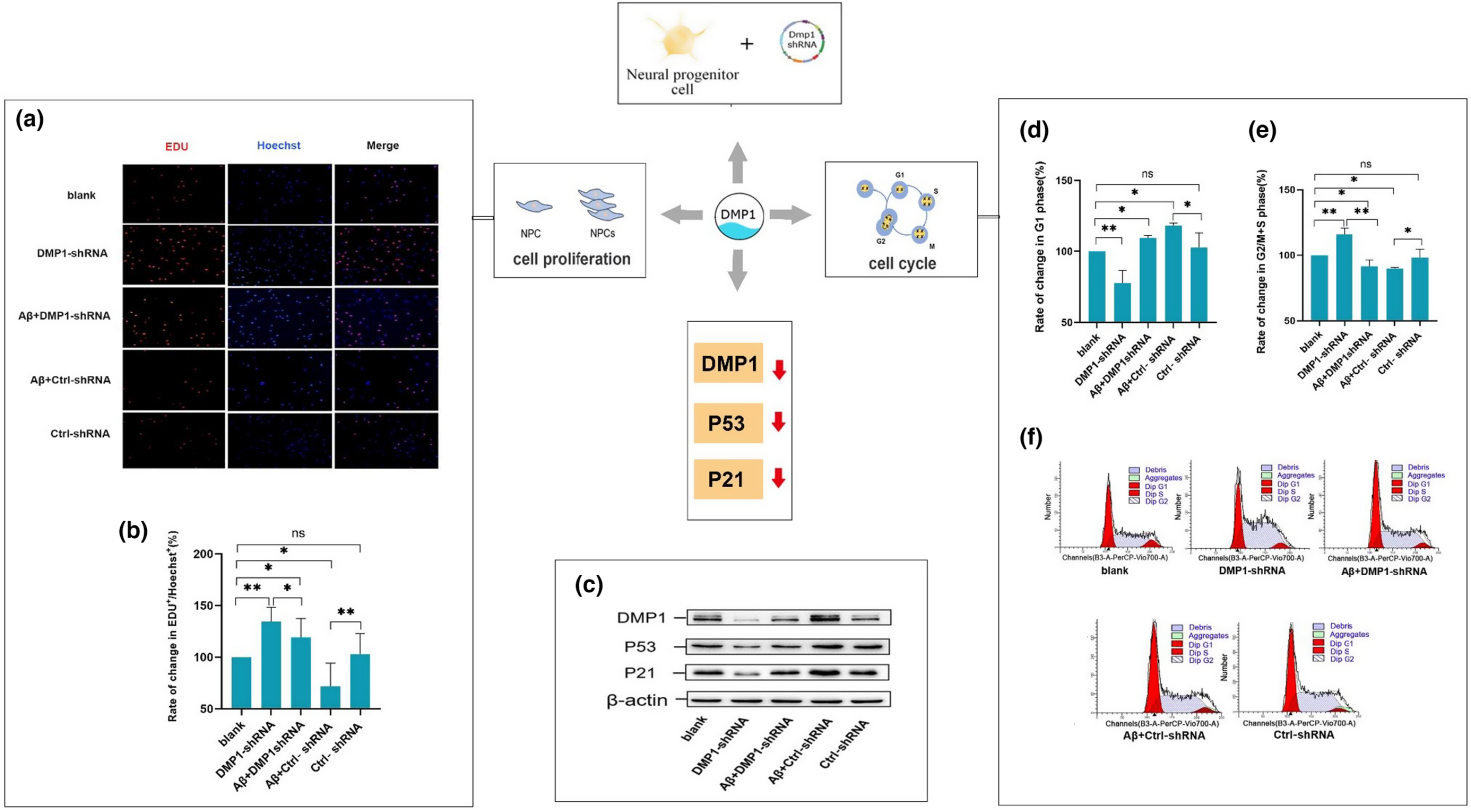
Effect of DMP1 knockdown on the proliferation of C17.2 neural progenitor cells. (a) Immunofluorescence photos of C17.2 cells. (b) Rate in change in EDU+/Hoechst+C17.2 cells. (c) The representative results of DMP1, P53, P21 and β‐actin expressed in the C17.2 cells in Western blot. (d) Rate of change in G1 phase in C17.2 neural progenitor cells. (e) Rate of change in G2/M+S phase in C17.2 neural progenitor cells. (f) At the indicated time point, DNA content of the cells were determined to analyze the distribution of cell‐cycle phase. The mathematical model MODFIT was used to calculate the proportions of cells at each cell‐cycle phase. Data information: The data were expressed as Mean ± SEM, *n* = 3 for each group, *p < 0.05, **p < 0.01, ***p < 0.001

To assess the function of DMP1 in the NPC cell cycle, lentivirus containing a specific shRNA is used to reduce DMP1 expression. The cells were divided into five groups: the blank group, the Ctrl‐shRNA group, the DMP1‐shRNA group, the Aβ+DMP1‐shRNA group, and the Aβ+Ctrl‐shRNA group. DMP1 expression was significantly reduced in the DMP1‐shRNA group, compared to the blank group and the Ctrl‐shRNA group (Figure 3c). These results suggested that DMP1 was successfully knockdown in the C17.2 neural progenitor cells.

The proliferation of the C17.2 neural progenitor cells was tested by EDU staining and FCM after DMP1 knockdown. Compared to the DMP1‐shRNA group, the amount of EDU^+^ and G2+S phase cells in the Aβ+DMP1‐shRNA group was significantly decreased (Figure 3a, b, e, f). These results suggested that DMP1 knockdown could improve the ability of C17.2 neural progenitor cells proliferation and cell‐cycle arrest induced by Aβ_1–42_.

Expressions of P53 and P21, were dramatically downregulated after DMP1 knockdown (Figure 3c). These data indicated that loss of the DMP1 expression resulted in the decreased P53 and P21 expression.

### 2.4 DMP1 overexpression impaired proliferation of C17.2 neural progenitor cells

**FIGURE 4:**
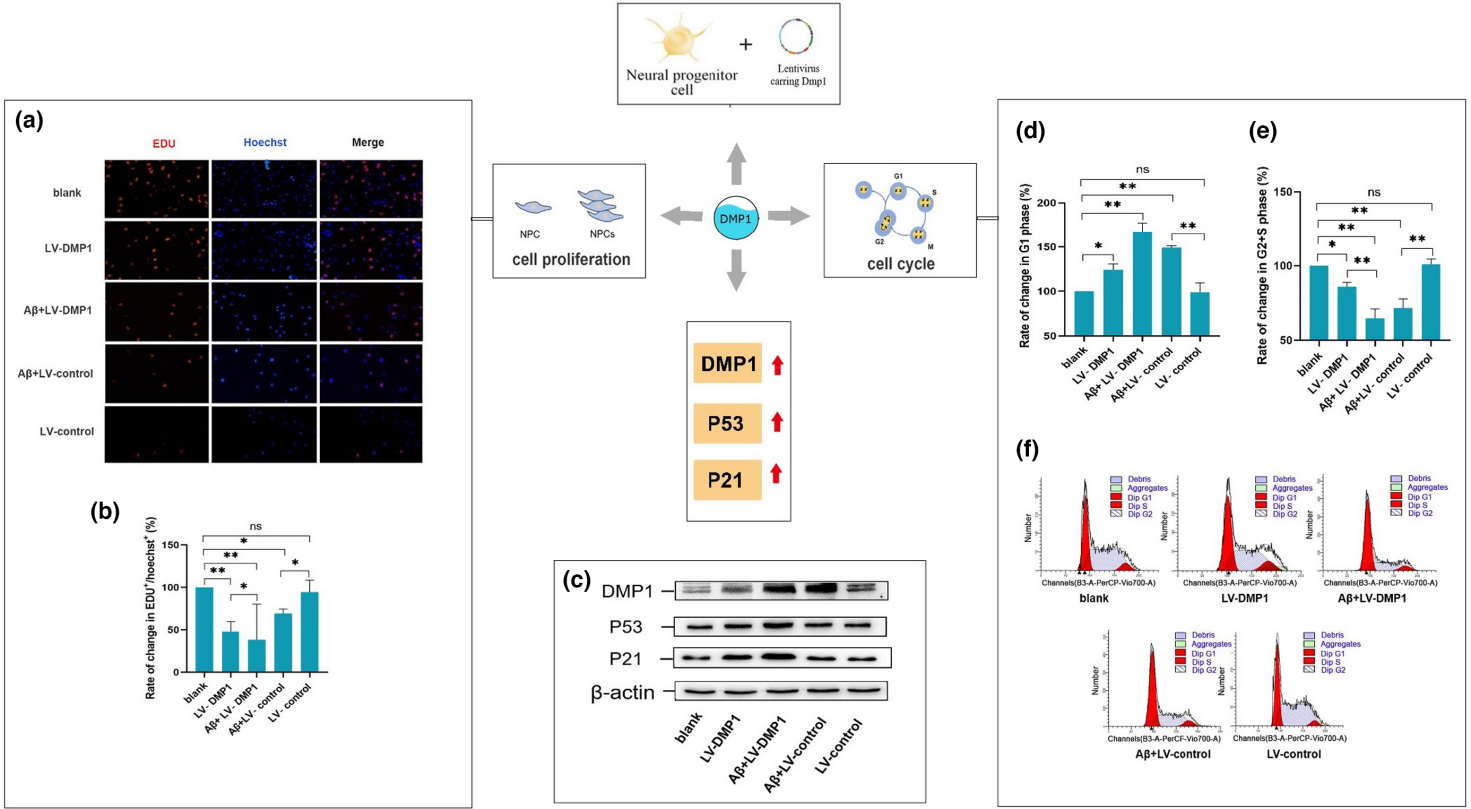
Effect of overexpressing DMP1 on the proliferation of C17.2 neural progenitor cells. (a) Immunofluorescence photos of C17.2 cells. (b) Rate in change in EDU+/Hoechst+C17.2 cells. (c) The representative results of DMP1, P53, P21 and β‐actin expressed in the C17.2 cells in Western blot. (d) Rate of change in G1 phase in C17.2 neural progenitor cell. (e) Rate of change in G2/M+S phase in C17.2 neural progenitor cells. (f) At the indicated time point, DNA content of the cells were determined to analyze the distribution of cell‐cycle phase. The mathematical model MODFIT was used to calculate the proportions of cells at each cell‐cycle phase. Data information: The data were expressed as Mean ± SEM, *n* = 3 for each group, *p < 0.05, **p < 0.01, ***p < 0.001

Next, the effects of DMP1 overexpression on the NPC cell cycle by transferring a lentiviral vector expressing DMP1 into the C17.2 neural progenitor cells was tested. Also, five groups were constructed with the cells: the blank group, the LV‐control group (a lentiviral vector contains control shRNA), the LV‐DMP1 group, the Aβ+LV‐DMP1 group, and the Aβ+LV‐control group. A significant upregulation of DMP1 expression in the LV‐DMP1 group compared to the LV‐control group was confirmed (Figure 4c), which suggested that DMP1 was successfully overexpressed in the C17.2 neural progenitor cells.

In addition, the impact of overexpressing DMP1 on the proliferation of C17.2 neural progenitor cells was evaluated by using EDU staining and FCM. Compared with the LV‐DMP1 group, the Aβ+LV‐DMP1 group accompanied by increased the number of G1 phase cells, while the number of EDU‐positive cells and G2+S phase cells was significantly decreased. Our data suggested that DMP1overexpression weakened the proliferative ability of C17.2 neural progenitor cells induced by Aβ_1–42_.

Furthermore, the levels of P53 and P21 were significantly induced after DMP1 overexpression (Figure 4c). Taken together, our data showed that overexpressing DMP1 lead to the increased expressions of P53 and P21.

### 2.5 The increased expression of DMP1 in the Alzheimer‐like mice

In our study, SAMP8 mice were selected as the AD model animals, SAMR1 mice are used as wild controls of SAMP8 mice. First, the protein levels of DMP1, P53 and P21 between the AD group (SAMP8) and the control group (SAMR1) were detected by western blot. The expressions of DMP1, P53 and P21 was significantly increased in the AD group (SAMP8) compared to the control group (SAMR1) (*p* < 0.01, Figure 6e–g). Beyond that, the number of hippocampal BrdU^+^/Nestin^+^ and BrdU^+^/DCX^+^ cells of the AD group (SAMP8) were significantly lower than those in the control group (SAMR1) (*p* < 0.01, Figure 6b, c), which suggested that the newborn NPCs and neurons decreased in Alzheimer‐like mice. In summary, the data revealed that DMP1 may play an important role in mediating the NPC proliferation in SAMP8 mice.

### 2.6 Silencing DMP1 promoted cognitive function in SAMP8 mice

To further verify that downregulation DMP1 on cognitive function improvement, animal experiments were conducted. To infect NPC across the blood‐brain barrier, intravenous injection of adenovirus‐shRNA‐DMP1 (AAV‐shRNA, 25 μl, PHP.EB) and adenovirus‐shRNA‐control (AAV‐GFP, 25 μl, PHP.EB) was to inhibit DMP1 expression in the SAMP8 mice of 5 months. The mice were divided into 4 groups: the control group (SAMR1), the AD group (SAMP8), the SAMP8+AAV‐GFP group and the SAMP8+AAV‐shRNA group. At 1month post‐infection, the impact of DMP1‐shRNA injection on cognitive functions was tested in SAMP8 mice. The efficiency of AAV‐shRNA virus‐mediated DMP1 knockdown was evaluated by western blot (presented in Figure 6d). Compared to the AD group (SAMP8), the DMP1 expression was significantly decreased in the SAMP8+AAV‐shRNA group (*p* < 0.01, Figure 6e); There was no difference in DMP1 expression between the AD group (SAMP8) and SAMP8+AAV‐GFP group (*p* > 0.05, Figure 6e). The silencing efficiency was 48.7%.

**FIGURE 5:**
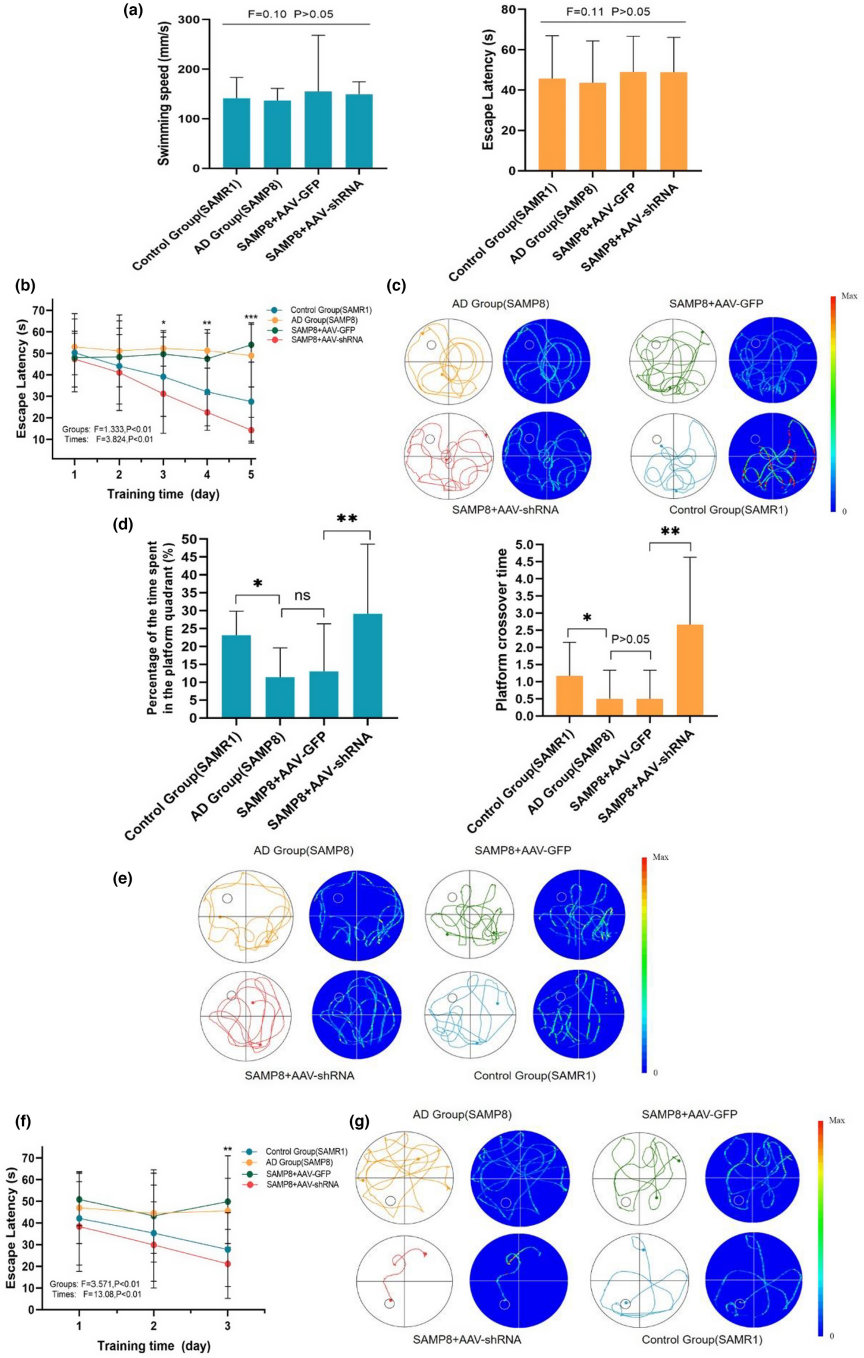
Silencing DMP1 in SAMP8 animals restores cognitive deficits. Morris water maze test was used to evaluate the learning and memory function of the mice. Escape latency in the formal experiments of the water maze task. (a) During the visible platform trail, there is no difference between the four groups in swimming speed and escape latency. (b) The escape latency of four groups in the hidden platform trail. (c) The heat maps of pooled animals manifest the results of the hidden platform trail. (d) Percentage of time spent in the platform quadrant and platform crossover time in the probe trail. (e) The heat maps of pooled animals manifest the results of the probe trail. (f) The escape latency of four groups in the reference trail. (g) The heat maps of pooled animals manifest the results of the probe trail. Data information: The data were expressed as Mean ± SEM, *n* = 5 for each group, *p < 0.05, **p < 0.01, ***p < 0.001

Cognitive function quantitatively was measured by using the Morris water maze (MWM) test, which is widely accepted as a behavioral paradigm for evaluating spatial working memory(Terry, 2009). We did not observe any difference on swim speed and escape latency during the visible platform trail (*p* > 0.05), which suggested that no significant difference in exercise ability was found among these groups. During hidden platform trail (5 days, platform was 1 cm hidden underwater, three trials per day and per animal), the data revealed that the escape latency was progressively decreased in the SAMP8+AAV‐shRNA group and the control group. The AD group (SAMP8) and the SAMP8+AAV‐GFP group had no difference in the escape latency (Figure 5b). On day 6, the DMP1 knockdown animals showed a significantly lower escape latency compared to the two groups with AD (*p* < 0.001) (Figure 5b). Furthermore, on day 7 of acquisition prior to the last 3‐d training, the probe trial was performed where the mice were swimming freely without a platform to escape. We compared the mean time in percentage of the time spent in the target quadrant and platform crossover time. Figure 5d shows that the SAMP8+AAV‐shRNA group had more platform crossover time and more time spent in the target quadrant compared to the SAMP8+AAV‐GFP group. The last test, the reversal trail, the location of platform was changed every day in the last 3‐d training session. The escape latency was also used as the evaluation criteria. On the last training day, the SAMP8+AAV‐shRNA group showed a significantly lower escape latency compared to the two groups with AD (*p* < 0.01) (Figure 5f). The results of MWM test revealed significant differences between SAMP8 mice injected with DMP1‐shRNA and the normal SAMP8 mice. Taken together, these data indicated that DMP1 knockdown ameliorated impaired spatial memory in SAMP8 mice.

### 2.7 Loss of DMP1 expression increased the number of NPCs in the hippocampal of SAMP8 mice

To identify the proliferation of endogenous NPC after silencing DMP1, 6month mice were administered 100mg/kg BrdU three times a day for last three consecutive days before mice were killed. The brain sections were incubated with the neural progenitor cell marker Nestin and the proliferation marker BrdU and then detected by immunostaining. The number of BrdU^+^/Nestin^+^ cells of SAMP8 and SAMP8+AAV‐GFP groups were significantly lower than those in the control and the SAMP8+AAV‐shRNA groups in the dentate gyrus. (Figure 5b, *p* < 0.05). Furthermore, we detected the number of newborn neurons in hippocampal. The number of newborn neurons was significantly decreased in SAMP8 and SAMP8+AAV‐GFP groups compared to the control and SAMP8+AAV‐shRNA groups (Figure 5c, *p* < 0.01). Therefore, silencing DMP1 could rescue memory impairments and NPC proliferation disorders in SAMP8 mice.

### 2.8 Inhibition of DMP1 expression resulted in the downregulation of P53 and P21

**FIGURE 6:**
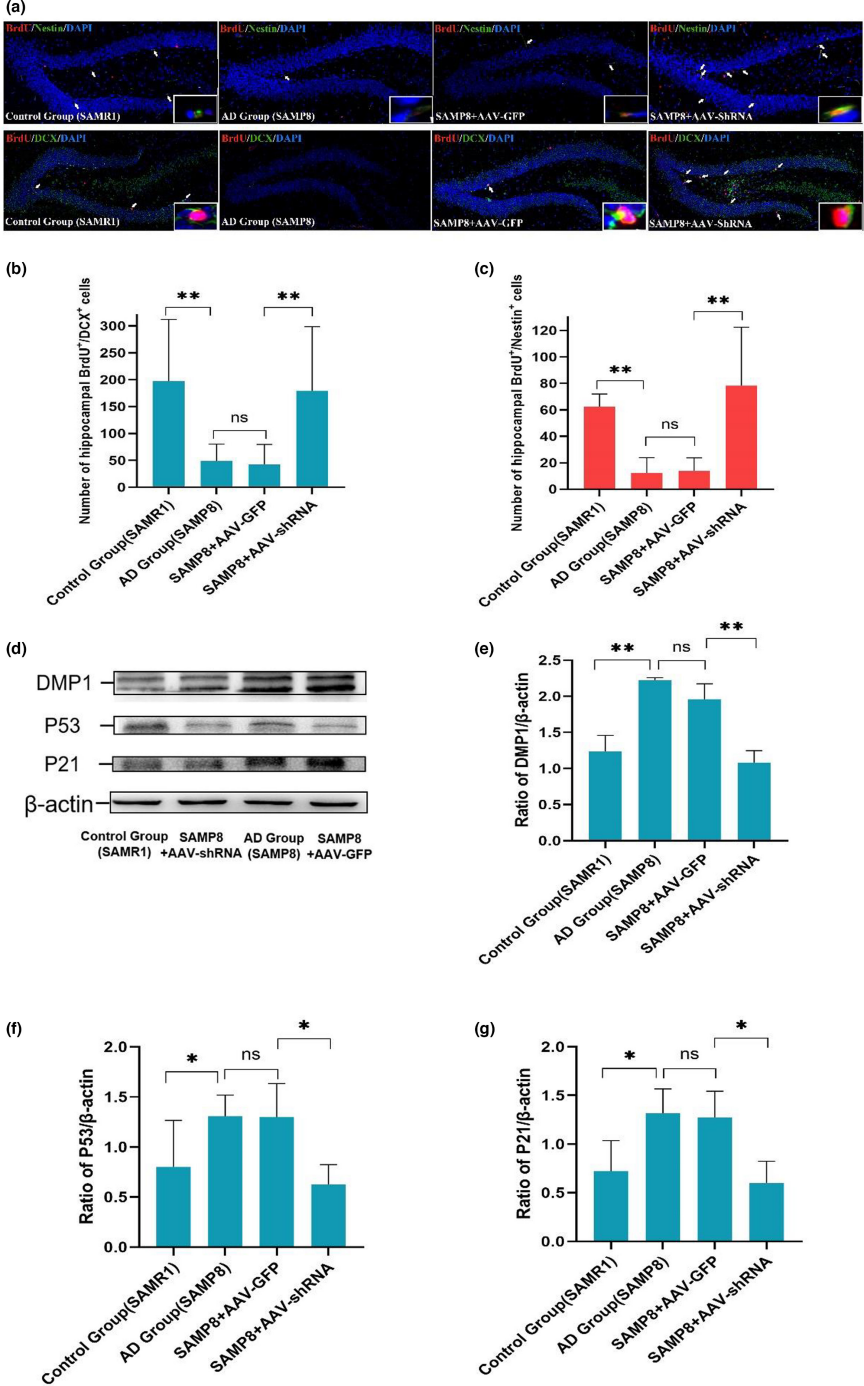
Silencing DMP1 promoted the number of hippocampal newborn NPCs and neurons through P53/P21 signaling in 6 months SAMP8 mice. Newborn NPCs in the hippocampus were detected by immunofluorescent staining with antibody against BrdU and Nestin, newborn neurons in the hippocampus were detected by immunofluorescent staining with antibody against BrdU and DCX. (a)–(c) Quantitative analysis of the number of BrdU^+^/Nestin^+^ and BrdU^+^/DCX^+^ cells. (d) The protein levels of DMP1, P53, and P21 were analyzed by western blot. (e)–(g) Quantitative analysis of proteins expression. Data information: The data were expressed as Mean ± SEM, *n* = 3 for each group, *p < 0.05, **p < 0.01, ***p < 0.001

We also detected alterations in P53 and P21 expressions in the hippocampus of SAMP8 mice, SAMP8+AAV‐GFP and SAMR1 groups. Compared to the SAMP8+AAV‐shRNA group, we found that P53 and P21 expression was dramatically downregulated in the SAMP8+ AAV‐shRNA group (*p* < 0.05, Figure 6d). Taken together, these data suggested that inhibition of DMP1 expression followed by the decreased P53 and P21 expression.

## 3 DISCUSSION

DMP1 plays a role in providing cell autonomous tumor surveillance, which bring about the senescence or apoptosis of cancer cells to prevent the development of cancer(Maglic et al., 2013). Our data suggested that DMP1 is involved in the cell‐cycle arrest of NPC induced by Aβ_1–42_. In this study, we investigated the clinical SNP data, which showed that DMP1 is closely correlated with cognitive function. The negative role of DMP1 on the proliferation of NPC was revealed by silencing and overexpressing DMP1 in vitro. Reduced DMP1 expression results in a decrease in P53 and P21 expression, which in turn leads to increased newborn NPC and neurons in the SAMP8 mice. This study showed that silencing DMP1 could improve cognitive dysfunction in SAMP8 mice. This improvement may be through enhancing the proliferation of NPCs Our finding provides a promising route toward ameliorating cognitive decline.

Transcriptomics studies suggest that the upregulated expression of DMP1 in AD model animals (Hokama et al., 2014). Furthermore, a significant association between the DMP1 SNPs and cognitive function was revealed by our clinical genomic analysis of ADNI database. There were 809 samples that met the enrollment requirements. However, we found that the majority of ADNI participants contained two or more miscellaneous SNPs. It's hard to evaluate the relation between a single DMP1 SNP and cognitive function. To address the above issue, LASSO regression is used to merge multiple SNPs into four clusters: exacerbating SNPs, protective SNPs, relevant SNPs (it has both exacerbating SNPs and protective SNPs) and irrelevant SNPs. To avoid potential statistical bias, clusters of exacerbating SNPs and protective SNPs were excluded for few samples. In parallel, we compared the distribution of each cluster among healthy individuals, MCI patients and AD patients, but no significant difference was found among the three groups. These data suggested that the seven DMP1 SNPs (relevant SNPs) located in the intro region are not strongly associated with the onset of AD, but they may affect cognitive function via regulation of DMP1 gene expression. Previous research also found that SNPs in the intron region were associated with gene expression(Lamba et al., 2008). Based on these, AD patients with different DMP1 SNPs may show variable DMP1 expression levels. According to our analysis of the clinical data, people with different DMP1 SNPs showed different levels of cognitive function. We further speculate that the expression of DMP1 is likely to affect cognitive function. Meanwhile, our data showed that the increased DMP1 protein expression had a relationship with the cognitive function in AD model mice.

DMP1 is a transcription factor which can induce cell‐cycle arrest of multiple cell types (Frazier et al., 2012; Inoue et al., 2007). DMP1 can be activated by RAS, which is a central signal transducer, functioning as a GDP/GTP‐regulated molecular switch (GTPase). RAS transformation usually brings out enhanced c‐Jun transcriptional activity and increased AP‐1‐mediated gene expression. c‐Jun is the most prominent AP‐1 protein that can activate the Dmp1 promoter to upregulation the expression of DMP1 in response to oncogenic Ras‐Raf signaling (Sreeramaneni et al., 2005). We found that Aβ_1–42_ lead to the increased expression of DMP1 in the NPC, which, in turn, impacted P53/P21 expression. Present results suggested that the NPC proliferation may be mediated by the DMP1/P53/P21 pathway. Through the carboxyl‐terminus of P53 and the DNA‐binding domain of DMP1, DMP1 and P53 can interact directly in mammalian cells, then DMP1 antagonized P53 ubiquitination and promoted P53 nuclear localization (Braithwaite et al., 2006; Frazier et al., 2012; Li et al., 2003). Mechanistically, the co‐expression of DMP1 and P53 could induce the expression of P53 target genes (Frazier et al., 2012). P53 participates in the process of cell‐cycle arrest through transcriptional activation of P21(Jung et al., 2010), activated P21 then inhibits cyclins and CDKs (Gartel et al., 1996). We found that DMP1 might induce NPC proliferation inhibition and G1 phase arrest. Our animal experiments suggested that silencing DMP1 may improve cognitive function. Silencing DMP1 by AAV only has brain tissue specificity, which could theoretically increase the number of all cells with proliferation potential including both NPC and glial cells. The present data showed that silencing DMP1 in SAMP8 mice increased the number of BrdU^+^/Nestin^+^ and BrdU^+^/DCX^+^ cells, which indicated that newborn neural progenitor cells and neurons increased in the hippocampal of SAMP8 mice. Whereas the sole origin of newborn neuron is the neural progenitor cell located in the SGZ and SVZ regions of mammalian brain (Fares et al., 2019). Newborn functional neurons produced by NPCs affect cognition through integrating into pre‐existing neural networks (Abbott & Nigussie, 2020). Meanwhile, previous studies illustrated that the glial cells involved influencing existing neurons and further indirectly impacted cognitive processes (Adamsky et al., 2018; Orr et al., 2015; Santello et al., 2019). As newborn neurons play central role in cognitive function, our results suggested that silencing DMP1 improved cognitive function through enhancing NPC proliferation.

Recent studies favor that adult hippocampal neurogenesis exists through human whole life (Tobin et al., 2019). Two regions of the mammalian brain, the dentate gyrus (DG) of the hippocampus and the subventricular zone (SVZ), are considered as the main regions for adult neurogenesis occurrence (Zhao et al., 2008). It is demonstrated that adult hippocampal neurogenesis has a direct effect on cognitive function for two reasons (Anacker & Hen, 2017; Poulose et al., 2017; Tobin et al., 2019). First, the hippocampal region has been shown to be the most affected brain region in AD (Armstrong & Cairns, 2015; Bekinschtein et al., 2010). Second, the adult newborn neurons that have enhanced synaptic plasticity are involved in hippocampus‐dependent learning and memory (Clelland et al., 2009; Sahay et al., 2011; Schmidt‐Hieber et al., 2004). It is demonstrated that the hippocampal neurogenesis moderately declines with aging resulted in decreasing the number of neuroblasts sharply (Deng et al., 2010; Schmidt‐Hieber et al., 2004; Tobin et al., 2019). Moreover, a recent research discovered a decline in the number of DCX^+^PCNA^+^ neuroblasts, which may be correlated with ameliorative cognitive scores and clinical diagnosis in people with MCI (Tobin et al., 2019). Besides the above effects, several factors may affect the cognitive function via neurogenesis, such as aging, neuroinflammation, oxidative stress, and brain injury (Poulose et al., 2017). Among those, an interesting phenomenon occurred: adult neurogenesis is a process that associated with ROS accumulation and immoderate oxidative stress (Le Belle et al., 2011). Concretely, Calabrese et al suggested that different exposure durations to ROS‐producing agent had different results: the short‐term exposure increased cell proliferation, while the long‐term incubation induced cellular apoptosis termed as hormesis paradigm (Calabrese, Cornelius, Dinkova‐Kostova, et al., 2010; Yuan et al., 2015). Several chemicals such as oxidizable diphenols, sulforaphane, dimethyl fumarate, celastrol, curcumin, nitric oxide (NO), carbon monoxide, hydrogen sulfide and so on, play important roles in hormetic‐based neuroprotection (Calabrese et al., 2010). The above hormetic agents emerged their roles in neuroprotection by against oxidative stress through sirtuin pathway, Keap1/Nrf2/ARE pathway and vitagenes network (Calabrese et al., 2007; Calabrese, Cornelius, Dinkova‐Kostova, et al., 2010; Calabrese, Cornelius, Maiolino, et al., 2010). Combined enhanced NPC proliferation with hormetic‐based neuroprotective therapies may achieve additional cognitive improvement.

Analysis of clinical genomics data suggested that the seven relevant DMP1 SNPs were closely related to cognitive function. In vitro study revealed the negative role of DMP1 on NPC proliferation by silencing and overexpressing DMP1. In vivo study showed that silencing DMP1 alleviate cognitive dysfunction of AD model mice through DMP1/P53/P21. Overall, DMP1 may be a promising target for overcoming memory deficits in AD.

## 4 MATERIALS AND METHODS

### 4.1 ADNI cohort

ADNI is a multisite, longitudinal, observational study that provides promising information on cognitive biomarkers, people with mild cognitive impairment (MCI), and patients with mild Alzheimer's disease (AD) since 2004. The overall goal of ADNI is to validate whether clinical biomarkers and neuropsychological assessment can be combined to measure the progression of the pathology of AD. Descriptive and demographic characteristics of ADNI participants are recorded and accessible. ADNI participants are divided into three classifications: AD group, MCI group, and normal aging group. The classifications can alter throughout time. 809 ADNI participants who have participated in whole genome sequencing were analyzed in this study.

### 4.2 Cognitive function test

In this study, the cognitive function of ADNI participants is evaluated by ADAS‐Cog. The cognitive subscale of the ADAS (ADAS‐cog) is a part of ADAS, which is used to grade the degree of the cognitive impairments. ADAS‐cog is considered as the cognitive measure standard for mild‐to‐moderate AD clinical trials (Rosen et al., 1984). Three key cognitive domains: language, memory, and praxis, which comprises 11 items of ADAS‐cog. The global ADAS‐cog score is in the 0–70 range, the higher score represents more cognitive impairment severity commonly associated with AD.

### 4.3 Whole genome sequencing

The Brin‐Wojcicki Foundation gave a favor on the support of Whole genome sequencing (Liu et al., 2021). The Alzheimer's Association was performed on 818 subjects from the ADNI Study by Illumina's non‐CLIA laboratory at roughly 30–40× coverage in 2012 and 2013. The variant calling workflow description below is composed of pre‐processing to analysis‐ready reads (BAM files) and variant calling. First, in order to make the data generated by the sequencers suitable for variant calling analysis, the data were put through several pre‐processing steps. The GATK Haplotype Caller was run on each sample's BAM file(s) to create single‐sample gVCFs. Next, data aggregation step is used to run CombineGVCFs on batches of ~200gVCFs to hierarchically merge a few hundred ADNI samples into a single gVCF. The next step is to create a set of raw SNP and indel calls. All the outputs were run by GenotypeGVCFs, this step is called Joint genotyping with all available samples. Finally, a machine learning method is constructed to assign a well‐calibrated probability in a raw call set. The variant quality score is used to filter the raw call set, thus produced a subset of desired quality calls, with the achievement of specificity and sensitivity.

### 4.4 Cell lines and cell cultures

The C17.2 neural progenitor cells was purchased from Bnbio Biotechnology (Beijing, China). For routine cultures, the C17.2 cells were seeded in 75 cm^2^ cell culture bottle. The medium components are H‐DMEM supplemented with 10% fetal calf serum, 100 U penicillin/ml and 100 μg streptomycin/ml. Using 0.05/0.02% trypsin/EDTA (KeyGEN Biotechnology, Nanjing, China) to digest the 80–90% confluent cells and seeded in a new cell culture bottle.

### 4.5 Aβ_1–42_ exposure

Aβ_1–42_ (Glbiochem, Shanghai, China) was dissolved in H‐DMEM medium for the 100 mM concentration. Before added to the cells, the Aβ_1–42_ stock solution was diluted to concentrations of 20 mM with H‐DMEM medium. The control group was exposed to medium without Aβ_1–42_ stock solution.

### 4.6 Western blot analysis

The original medium was removed, and then 12 well culture plate was washed once with PBS, and lysed with Western and IP lysate buffer (Beyotime Biotechnology, Shanghai, China). The proteins’ concentration was detected by Bradford method (Bradford protein assay kit; YEASEN Biotechnology), SDS‐polyacrylamide gel electrophoresis was used to separate proteins and then transferred the proteins onto polyvinylidene difluoride membranes (Millipore, Burlington, MA, USA). After blocking in blocking buffer (Beyotime Biotechnology, Shanghai, China), primary antibodies incubated the PVDF membrane for 18h (rabbit anti‐β‐actin 1:3000, rabbit anti‐DMP1 1:2000, mouse anti‐P53 1:1000, rabbit anti‐RAS 1:2000; Cell Signaling Technology [CST]), (rabbit anti‐P21 1:1000; Abcam). ECL western blotting detection reagents (Tannon ScienceI & Techonlogy, Shanghai, China) were used to detect the immunoreactivity. The results were tested by a gel image system. The results were analyzed by the Image J software.

### 4.7 EDU incorporation and staining

The EDU kit was purchased from Beyotime Biotechnology (Shanghai, China). Preparation of 2X EDU solution: 1:500 dilution with culture medium. Firstly, adding EDU solution into the culture plate and incubated for 2 h. After labeling, the cells were fixed in 100 μl of 4% formaldehyde for 15 min at room temperature (RT) and washed 3 times. Next, the cells were incubated with Triton X‐100 for 15 min at RT and washed 3 times. For the EDU click reaction, the cells were treated with click solution, incubated for 30 min at RT in dark place, and then washed 3 times with PBS. After that, the cells were treated with Hoechst 33342, incubated for 10 min at RT in the dark and washed 3 times. Inverted fluorescence microscope (Olympus Corporation, Japan) was used to observe the cells.

### 4.8 FCM analysis of cell cycle

The analysis of cell cycle was measured by cellular DNA content with FCM. Cell‐cycle kit was purchased from Fcmacs Biotechonlogy (Nanjing, China). Briefly, adherent cells (90% confluence) were collected with accutase (Fcmacs Biotechonlogy, Nanjing, China) and centrifuged at 300g, then fixed in iced 75% ethanol for 16 h at 4℃. Fixed cells were centrifuged at 300g, washed once in iced PBS, and then resuspended in propidium iodide (PI) stain buffer (0.4 ml, 15 μl 25XPI, and 4 μl 2.5 mg/ml DNase‐free RNase A for 30 min warm bath in the dark. After staining, FCM was used to analyze the samples (Miltenyi Biotechonlogy, MACS Flow Cytometry, Germany) and data were analyzed with Modifit LT software.

### 4.9 Lentiviral transfection

Lentivirus carrying DMP1‐shRNA (DMP1‐shRNA; 5′‐CCTAAGGATAGCTGAGCTT‐3′) and control lentivirus (Ctrl‐shRNA; 5′‐TTCTTCGAACGTGTCACGT‐3′) were designed by Hanbio Biotechnology (Shanghai, China). Also, lentivirus to overexpress DMP1 (LV‐DMP1) and control lentivirus were established by Hanbio Biotechnology (Shanghai, China). After cells reached in 30–50% confluence, C17.2 neural progenitor cells were seeded in 24‐well culture plates and infected with the specific lentivirus (MOI  = 30). After transfection 24 h, the 24‐well culture plate medium was changed with normal medium. Transfection efficiency was measured by fluorescence microscopy after transfection 72 h (Olympus Corporation, Japan). To select the cells that successfully infected the virus, 8 μg/ml puromycin was added to the normal medium for 48 h. Later can be regular puromycin screening. The verification of efficiency of DMP1 knockdown and overexpression was measured by Western blotting.

### 4.10 Animals and animal grouping

Male SAMP8 and SAMR1 mice were used for this study, the mice were purchased from the Beijing HFK Bioscience Corporation (Beijing, China). The SAMP8 mice have certain behavioral abnormalities, which are similar to AD patients. The mice were feeded with food and water. Three groups were designed with 5 months SAMP8 mice, including the AD group (SAMP8), the SAMP8+AAV‐GFP group, and the SAMP8+AAV‐shRNA group (*n* = 6 for each group). The age‐matched SAMR1 mice (*n* = 6) were used as normal controls.

### 4.11 Morris water maze

The Morris water maze was constructed to evaluate the spatial memory of SAMP8 and SAMR1 mice(Zheng et al., 2021). Briefly, the swimming tank water temperature was maintained between 30°C and 37°C, edible melanin was poured into the water to make opaque. On the first day, the test was designed to see whether the mice could find a 1cm platform above the water surface in 60 s (visible platform trail). Then the mice were trained to find a 1 cm hidden platform underwater up to 60 s in five consecutive days. Each mouse was trained for three times per day. The position of mice entry into water was changed from three different quadrants. After every training session, each mouse was remained on the platform for 10 s. The tracks in the water of mice were kept a record by a behavioral instrument. On the sixth day, mice were given free from the opposite quadrant to find the position of the removed platform within 60 s (probe trial). There were several indicators needed to be recorded: the latency to reach the platform, the percent time spent in the target quadrant, the crossing times to the platform regions, and the swimming speed. After that, the mice received another three successive days training. During a three‐day spatial memory test, mice were released in the quadrant opposite the platform, and the platform position was changed each day. The latency to reach the platform was analyzed. Each mouse was tested three times a day.

### 4.12 BrdU incorporation and staining

SAMP8 mice were injected intraperitoneally with 50 mg/kg/day BrdU (Beyotime Biotechnology, Shanghai, China) for continuous three days before sacrificed. In turn, the slices were washed with xylene 15 min, anhydrous ethanol 5min, anhydrous ethanol 5 min, 85% alcohol 5 min, 75% alcohol 5 min, distilled water. Brain sections were placed in a repair box filled with EDTA antigen‐repair buffer (pH8.0) in a microwave oven for antigen‐repair. Medium fire for 8 min to boiling, then low fire for 7min. After natural cooling, the glass slides were placed in PBS (pH7.4) and washed by shaker for 3 times, 5 min each time. Then the sections were blocked for 30 min. Gently shake off the sealing solution, add PBS to the slices with a certain proportion of primary antibody (BrdU 1:300, DCX 1:300, Nestin 1:300, Servicebio, Wuhan) and incubated overnight at 4°C. The slides were placed in PBS (pH7.4) and washed by a shaker for 3 times, 5 min each time. The sections were incubated with the secondary antibody (1:300, Servicebio, Wuhan) at RT for 50 min in the dark. The slices were dried slightly and DAPI was added, incubated at RT for 10 min in the dark. The slides were placed in PBS (pH7.4) and washed by a shaker for 3 times, 5 min each time. Spontaneous fluorescence quenching agent was added into the ring for 5 min, and then flushed with water for 10 min. Last, the slices were dried slightly and sealed with anti‐fluorescence quenching sealing tablets. Sections were observed under fluorescence microscope (Nikon, Japan) and images were collected. Fluorescence was analyzed by the Indica Labs software.

### 4.13 Adenovirus transfection

Genomeditech Biotechnology (Shanghai, China) constructed the adenoviruses. The adenoviruses contained blank adenoviral vectors, and a DMP1 interfering sequence with green fluorescent protein or green fluorescent protein (AAV‐GFP) alone. The DMP1 short hairpin RNA (shRNA) targeting sequence was: 5′‐CCTAAGGATAGCTGAGCTT‐3′. The control ShRNA targeting sequence was 5′‐TTCTCCGAACGTGTCACGT‐3′. Adenovirus was injected through the caudal vein. The viral dose was 1.17^E+13^ VG/ml in 25 µL. Four weeks later, DMP1 protein expression levels were examined by western blotting to verify adenovirus transfection efficiency.

### 4.14 Statistical analysis

The forms of data are presented descriptively as the mean ± SEM. SPSS 22.0 and Prism 8.0 were used to analyze the data. Using an unpaired Student's *t* test assessed the difference between the two groups. One‐way ANOVA test was used to compare the means among multiple groups. All experiments were repeated three times, and *p* < 0.05 was considered statistically significant. In this study, clusters related to ADAS‐cog were selected by LASSO regression. LASSO regression is considered as a popular technique for feature selection, which can continuously shrinks coefficients. To reach the goal of dropping factors, LASSO shrinks some of coefficients to zero.

## 5 COFLICT OF INTEREST

The authors declare that they have no known competing financial interests or personal relationships that could have appeared to influence the work reported in this paper.

## AUTHOR CONTRIBUTIONS

H.Z., H.L. and X.L. designed the experiments. H.Z., J.W., Y.D., and P.C. performed the molecular biological experiments and animal experiments, H.Z. wrote this manuscript. All authors have read and agreed to the published version of the manuscript.

## Supporting information

Supplementary Material
**Table S1** Participants in ADNI cohort
Table S2 The SNP ID of four clusters classified by Lasso regression
**Figure S1** Alteration of protein expressions in RAS/DMP1/P53/P21 pathway induced by Aβ_1–42_. (a) Different Aβ_1–42_ exposure time resulted in the alteration of RAS expression between the control group and the Aβ group. (*n* = 3 for each group, Aβ_1–42_ exposure time: 2, 4, 6, 8, 10, 12, 18, 24 h). (b) Different Aβ_1–42_ exposure time resulted in the alteration of DMP1 expression between the control group and the Aβ group. (*n* = 3 for each group, Aβ_1–42_ exposure time: 2, 4, 6, 8, 10, 12, 18, 24 h). (c) Different Aβ_1–42_ exposure time resulted in the alteration of P53 expression between the control group and the Aβ group. (*n* = 3 for each group, Aβ_1–42_ exposure time: 2, 4, 6, 8, 10, 12, 18, 24 h). (d) Different Aβ_1–42_ exposure time resulted in the alteration of P21 expression between the control group and the Aβ group. (*n* = 3 for each group, Aβ_1–42_ exposure time: 2, 4, 6, 8, 10, 12, 18, 24 h)
**Figure S2** Cell cycle alteration of C17.2 neural progenitor cells induced by Aβ_1–42_. Different Aβ_1–42_ exposure time resulted in cell cycle alteration of C17.2 neural progenitor cel between the control group and the Aβ group. (*n* = 3 for each group, Aβ_1–42_ exposure time: 2, 4, 6, 8, 10, 12, 14, 16, 18, 20, 22, 24 h)

## Data Availability

All the necessary data that used in this manuscript has showed in the supplementary files. Besides, this manuscript does not contain the source data file. The source data generated during and/or analysed during the current study are available from the corresponding author upon reasonable request.

## APPENDIX A

Data used in preparation of this article were obtained from the Alzheimer's Disease Neuroimaging Initiative (ADNI) database (adni.loni.usc.edu). As such, the investigators within the ADNI contributed to the design and implementation of ADNI and/or provided data but did not participate in analysis or writing of this report. A complete listing of ADNI investigators can be found at: http://adni.loni.usc.edu/wpcontent/uploads/how_to_apply/ADNI_Acknowledgement_List.pdf.
