# Supplementary material for: Improved cognitive impairments by silencing DMP1 via enhancing the proliferation of neural progenitor cell in Alzheimer‐like mice
Source: Aging Cell. 2022 Apr 2;21(5):e13601. doi: 10.1111/acel.13601 (PMC9124312; doi:10.1111/acel.13601)
Supplement: Supplementary file 1 — Supplementary Material Table S1 Participants in ADNI cohort Table S2 The SNP ID of four clusters classified by Lasso regression Figure S1 Alteration of protein expressions in RAS/DMP1/P53/P21 pathway induced by Aβ1–42. (a) Different Aβ1–42 exposure time resulted in the alteration of RAS expression between the control group and the Aβ group. (n = 3 for each group, Aβ1–42 exposure time: 2, 4, 6, 8, 10, 12, 18, 24 h). (b) Different Aβ1–42 exposure time resulted in the alteration of DMP1 expression between the control group and the Aβ group. (n = 3 for each group, Aβ1–42 exposure time: 2, 4, 6, 8, 10, 12, 18, 24 h). (c) Different Aβ1–42 exposure time resulted in the alteration of P53 expression between the control group and the Aβ group. (n = 3 for each group, Aβ1–42 exposure time: 2, 4, 6, 8, 10, 12, 18, 24 h). (d) Different Aβ1–42 exposure time resulted in the alteration of P21 expression between the control group and the Aβ group. (n = 3 for each group, Aβ1–42 exposure time: 2, 4, 6, 8, 10, 12, 18, 24 h) Figure S2 Cell cycle alteration of C17.2 neural progenitor cells induced by Aβ1–42. Different Aβ1–42 exposure time resulted in cell cycle alteration of C17.2 neural progenitor cel between the control group and the Aβ group. (n = 3 for each group, Aβ1–42 exposure time: 2, 4, 6, 8, 10, 12, 14, 16, 18, 20, 22, 24 h) [file ACEL-21-e13601-s001.docx]

**Improved Cognitive impairments by silencing** **DMP1 via enhancing the proliferation of neural progenitor cell in Alzheimer-like Mice**

Authors: Huimin Zhao^1^, Jie Wei^1^, Yanan Du^1^, Peipei Chen^1^, Xiaoquan Liu^1,*^, Haochen Liu^1,*^

for the Alzheimer’s Disease Neuroimaging Initiative^**^

^*^ *Corresponds author*

Address:

*^1^ Center of Drug Metabolism and Pharmacokinetics, China Pharmaceutical University, Nanjing,* *210009, China*

Tel.: + 86-25-83271260

*E-Mail address: 3120010076@stu.cpu.edu.cn (HMZ); 1976614498@qq.com (JW); 1013577558@qq.com (YND);* *1191065374@qq.com (PPC);* *[lxq@cpu.edu.cn](mailto:lxq@cpu.edu.cn),* *haochenliu@cpu.edu.cn (XQL,* *HCL Corresponds author).*

**Improved Cognitive impairments by silencing DMP1 via enhancing the proliferation of** **neural progenitor cell** **in Alzheimer-like Mice**

| Table S1 Participants in ADNI cohort | | | |  |
| --- | --- | --- | --- | --- |
|  | AD Group | MCI Group | Control Group | |
| Number | 48 | 480 | 281 | |
| Age | 75.51±9.23 | 72.29±7.46 | 74.62±5.57 | |
| Gender(male/female) | 18/30 | 280/200 | 136/145 | |
| ADAS-cog | 29.80±8.43 | 15.31±6.60 | 8.94±4.39 | |

Table S2 The SNP ID of four clusters classified by Lasso regression

| Clusters | SNP ID |
| --- | --- |
| Exacerbating SNPs | rs1058972, rs3789252, rs4728686 |
| Protective SNPs | rs4140847, rs4728685, rs6966613, rs6465094 |
| Relevant SNPs | rs1058972, rs3789252, rs4728686, rs4140847, rs4728685, rs6966613, rs6465094 |
| Irrelevant SNPs | rs4639442, rs3789251, rs2074757, rs765968, rs6952842, rs10228392, rs10257953, rs3747807, rs7800541, rs2108271, rs2108272, rs10263920, rs7810647, rs1558050, rs7793356, rs7776634 |

| 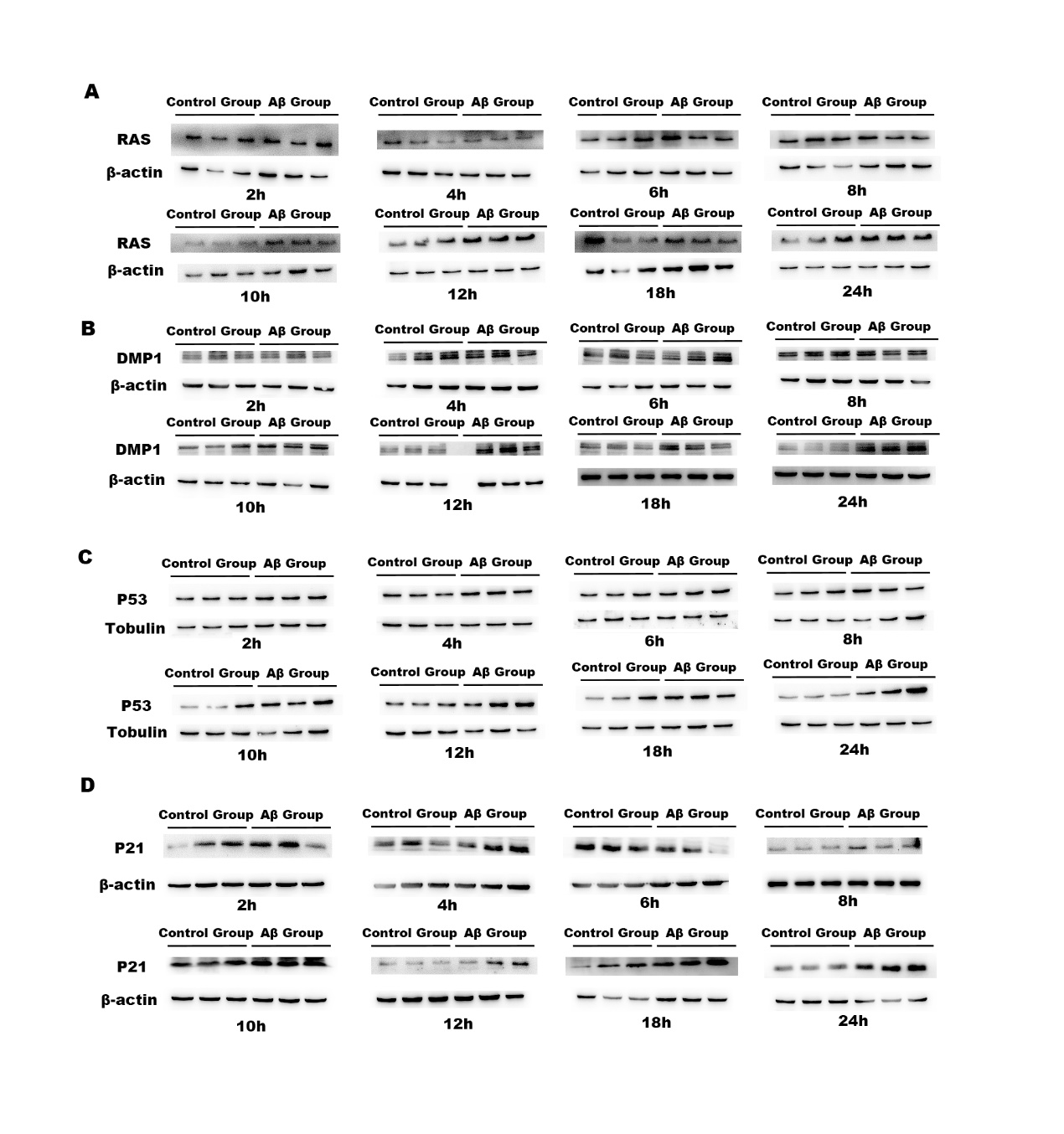 |
| --- |
| Figure S1. Alteration of protein expressions in RAS/DMP1/P53/P21 pathway induced by Aβ1-42  (A) Different Aβ1-42 exposure time resulted in the alteration of RAS expression between the control group and the Aβ group. (n=3 for each group, Aβ1-42 exposure time:2h,4h,6h,8h,10h,12h,18h, 24h)  (B) Different Aβ1-42 exposure time resulted in the alteration of DMP1 expression between the control group and the Aβ group. (n=3 for each group, Aβ1-42 exposure time:2h,4h,6h,8h,10h,12h,18h, 24h)  (C) Different Aβ1-42 exposure time resulted in the alteration of P53 expression between the control group and the Aβ group. (n=3 for each group, Aβ1-42 exposure time:2h,4h,6h,8h,10h,12h,18h, 24h)  (D) Different Aβ1-42 exposure time resulted in the alteration of P21 expression between the control group and the Aβ group. (n=3 for each group, Aβ1-42 exposure time:2h,4h,6h,8h,10h,12h,18h, 24h) |

| 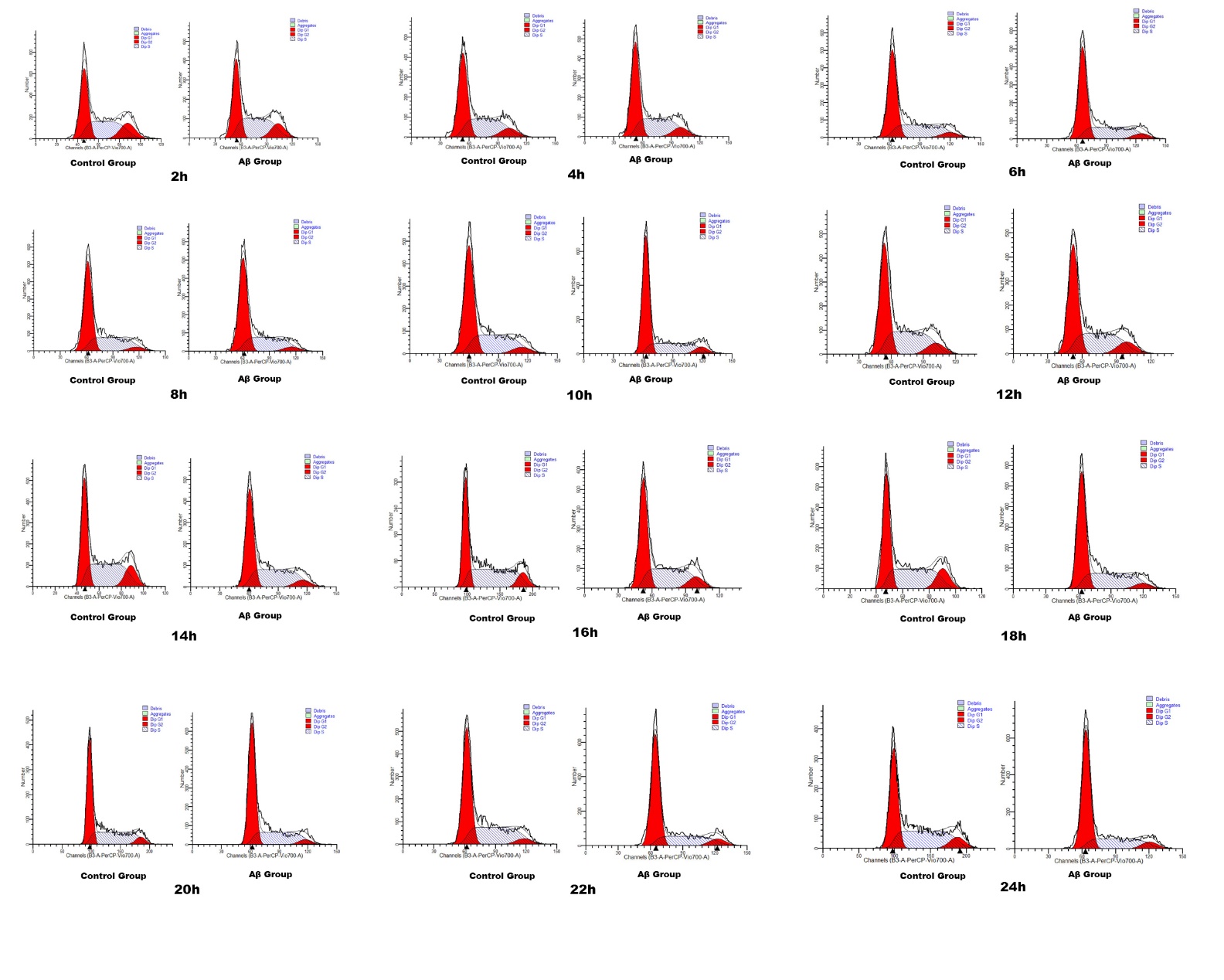 |
| --- |
| Figure S2. Cell cycle alteration of C17.2 neural progenitor cell induced by Aβ1-42  Different Aβ1-42 exposure time resulted in cell cycle alteration of C17.2 neural progenitor cel between the control group and the Aβ group. (n=3 for each group, Aβ1-42 exposure time:2h,4h,6h,8h,10h,12h,14h,16h,18h,20h,22h,24h.) |

**Figure S1** Alteration of protein expressions in RAS/DMP1/P53/P21 pathway induced by Aβ_1–42_. (a) Different Aβ_1–42_ exposure time resulted in the alteration of RAS expression between the control group and the Aβ group. (*n* = 3 for each group, Aβ_1–42_ exposure time: 2, 4, 6, 8, 10, 12, 18, 24 h). (b) Different Aβ_1–42_ exposure time resulted in the alteration of DMP1 expression between the control group and the Aβ group. (*n* = 3 for each group, Aβ_1–42_ exposure time: 2, 4, 6, 8, 10, 12, 18, 24 h). (c) Different Aβ_1–42_ exposure time resulted in the alteration of P53 expression between the control group and the Aβ group. (*n* = 3 for each group, Aβ_1–42_ exposure time: 2, 4, 6, 8, 10, 12, 18, 24 h). (d) Different Aβ1-42 exposure time resulted in the alteration of P21 expression between the control group and the Aβ group. (n=3 for each group, Aβ1-42 exposure time:2h,4h,6h,8h,10h,12h,18h, 24h)
